# From prenatal diagnosis to surgical treatment: two case reports of congenital granular cell epulis

**DOI:** 10.3389/pore.2024.1611834

**Published:** 2024-07-10

**Authors:** Yibing Han, Wen Qiu, Yu Zhang, Mengmeng Hua, Shaohua Liu, Zuoqing Dong

**Affiliations:** ^1^ Department of Oral and Maxillofacial Surgery, Qi Lu Hospital of Shandong University, Jinan, Shandong, China; ^2^ Department of Shandong Academy of Medical Sciences (SDAMS), Jinan, China; ^3^ Department of Pathology, Qi Lu Hospital of Shandong University, Jinan, Shandong, China

**Keywords:** case report, surgical treatment, congenital granular cell epulis, prenatal diagnosis, oral and maxillofacial regions

## Abstract

Herein, we detail a multidisciplinary approach and sequential treatment for two infants with congenital granular cell epulis (CGCE). Ultrasonic examinations at 34 weeks of gestation revealed prominent oral masses in both fetuses. To devise a carefully considered treatment strategy, a comprehensive multidisciplinary consultation including oral and maxillofacial surgeons, pediatricians, obstetricians, and anesthesiologists was convened. Following cesarean sections, the lesions were successfully removed, measuring approximately 30 × 15 mm and 30 × 20 mm in size, respectively. Immunohistochemical analysis showed that vimentin was positive, S-100 protein was negative, and NSE protein and CD68 protein were negative. These findings underscore the importance of prenatal diagnosis of congenital granular cell epulis for the effective management of these rare benign conditions.

## Introduction

Congenital granular cell epulis (CGCE), also referred to as congenital gingival granular cell tumour, congenital epulis or Neumann’ tumor [1–3], is a benign lesion that exclusively occurs in newborns, and has an incidence rate of approximately 0.0006% [4, 5]. The lesions frequently occur in the maxillary alveolar ridge, which is three times more common than in the mandibular alveolar ridge [6]. Females are affected 8 to 10 times more frequently than males [7]. The lesion typically appears as a solitary mass. Multiple lesions are sporadic and account for approximately 10% [8]. Clinically, the lesion presents as a lobular, sessile or pedunculated swelling with a smooth surface. Due to its origin in the oral cavity, large lesions may interfere with feeding and breathing. The histogenesis of the lesion remains unclear and controversial. Several hypotheses have been proposed regarding the origin of the lesion, including pericyte [6], fibroblast [9], histiocyte [10], nerve-related [11] and undifferentiated mesenchymal cells [12, 13]. The lesion may be diagnosed by ultrasound during pregnancy, especially during the third trimester [14]. Although spontaneous regression has been reported in the literature, surgical excision remains the most common treatment with negligible recurrence and malignant transformation [8].

In this article, we report on two cases of CGCE, which presented in the prenatal period with oral masses protruding from the mouth. We provide a detailed account of the prenatal diagnosis, clinical and histopathological characteristics, and the management of the lesions. This report underscores the significance of a sequential treatment approach for the lesions.

## Case series presentation

We have obtained the patient’s guardian’s informed consent for them to undergo surgery, as well as for the disclosure of clinical data, imaging, and other relevant information pertinent to this report.

### Prenatal diagnosis

**FIGURE 1:**
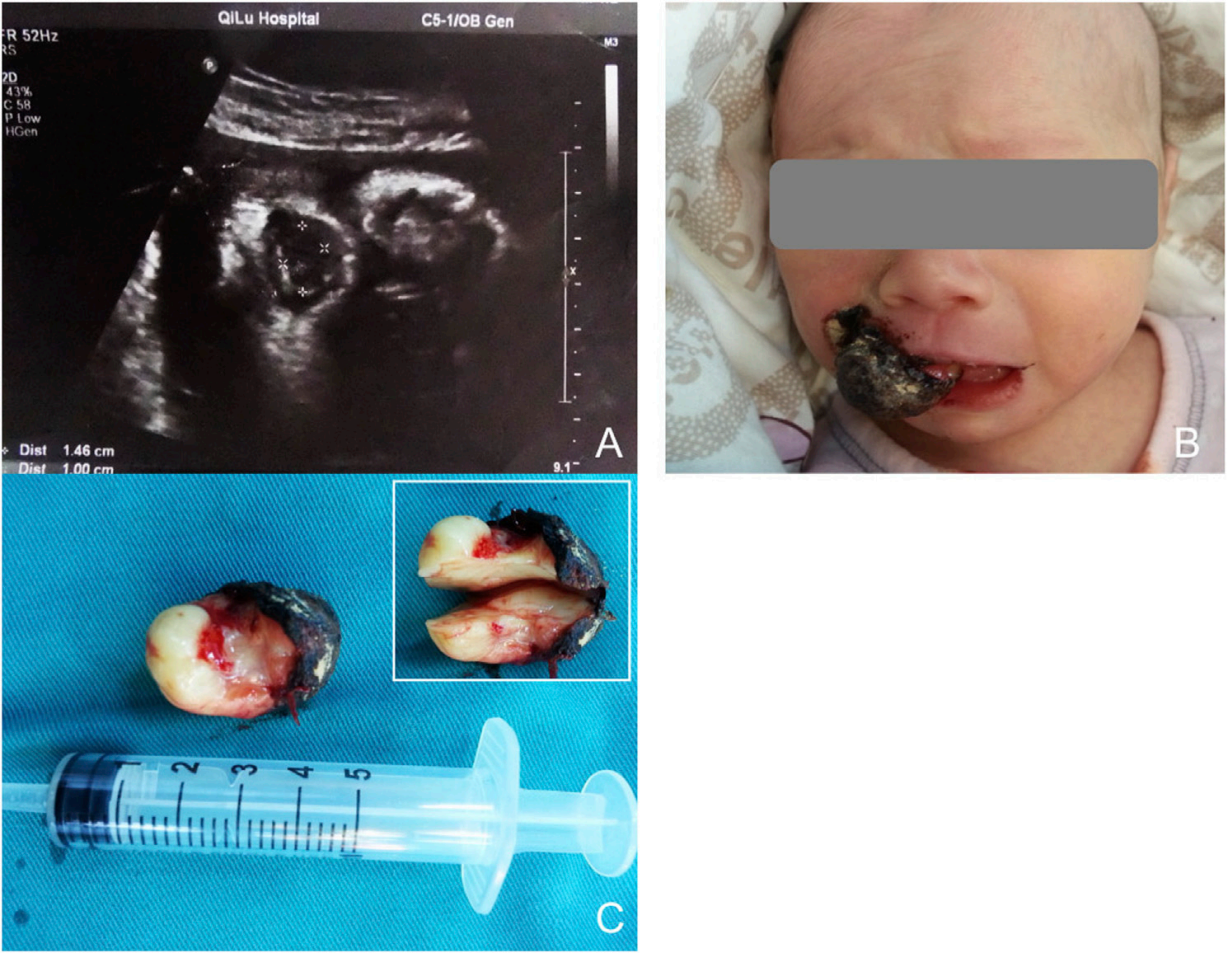
**(A)** Sonography at 34 weeks of gestion, showing a mass protruding from the mouth of the fetus. **(B)** Appearance of the baby with the oral mass. The surface of the lesion bled and formed ulceration. **(C)** The general specimen of the lesion. Up-right shows the cross section was yellowish-white and homogenous.

The first case was detected during an ultrasound examination at 34 weeks of pregnancy. The mother, who was 25 years old, had undergone previous normal ultrasounds at 12 and 22 weeks. A solid mass located in the maxillary part of the mouth was detected, it measured about 15 mm × 10 mm (Figure 1A). The initial prenatal diagnosis suggested a congenital teratoma rather than CGCE.

**FIGURE 2:**
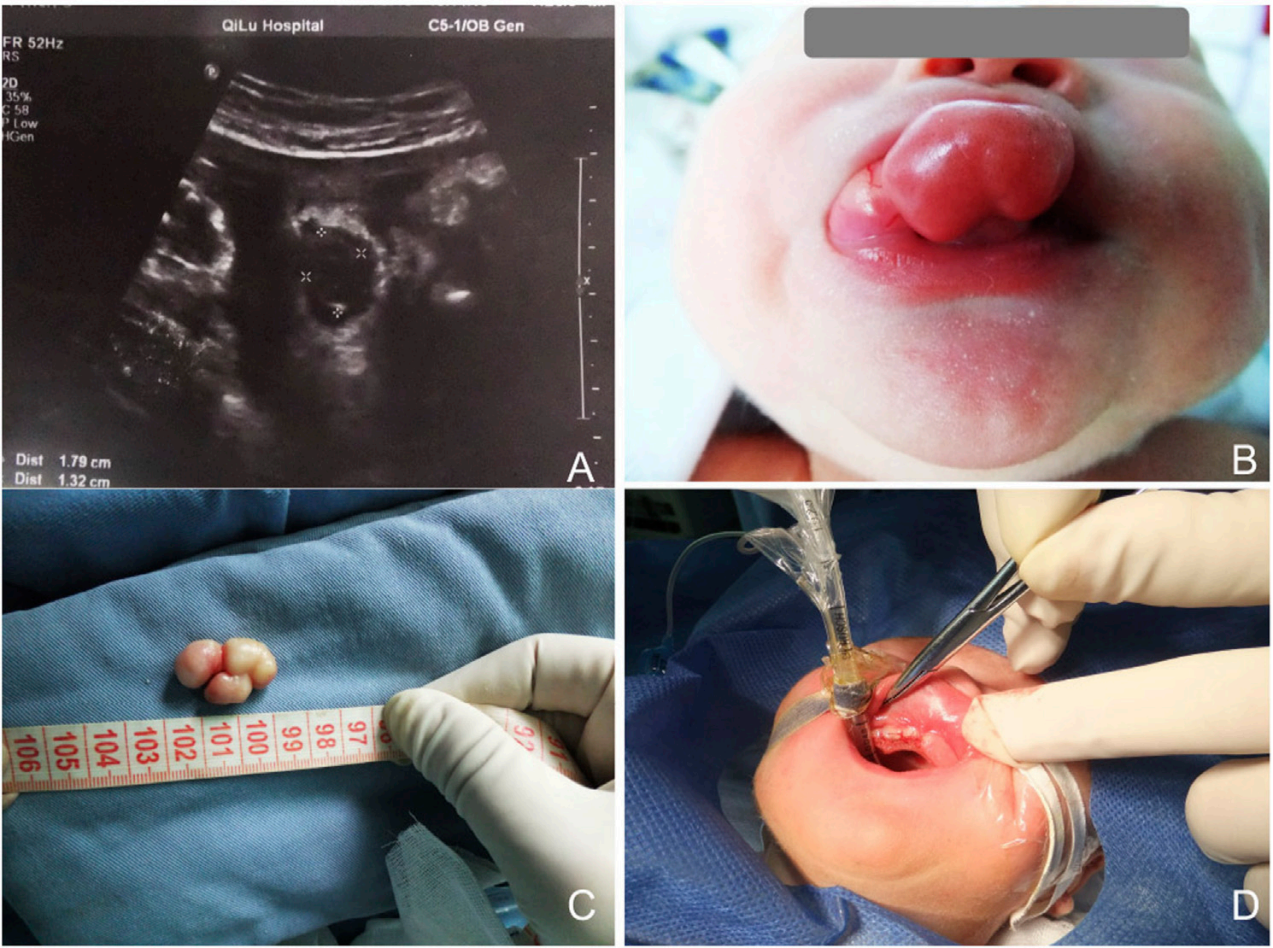
**(A)** Sonography at 34.5 weeks of gestation, showing a mass protruding from the mouth of the fetus. **(B)** Appearance of the baby with the oral mass. The lesion was sessile and lobular, with a smooth surface. **(C)** Gross view of the lesion. **(D)** Three absorbable sutures were used to close the wound.

The second case occurred 6 months later, during a routine ultrasound examination at 34 and a half weeks of pregnancy. The mother, aged 28, had previously undergone normal ultrasound scans. The mass, measuring approximately 18 mm × 13 mm, was again located in the maxillary region (Figure 2A). CGCE was primarily diagnosed based on the findings in the first case.

Both mothers were primiparae and had no history of miscarriage. They remained stable during pregnancy and had normal amniotic fluid. No medication was taken during pregnancy. Neither were married to close relatives and neither had genetic diseases or similar oral mass in their relatives (Table 1).

**TABLE 1:** Overview of prenatal diagnosis to treatment.

Sequence examination and treatment	Timeline	Case1	Case2
Pregnant week	Mother’s age	25y	28y
	12 week	sonographic anomalies	
	22 week		
	34 week	• Solid mass in the oral cavity • Size:15 * 9 mm	• Maxillary solid mass • Size:17 * 12 mm
Initial diagnosis	Congenital teratoma		Congenital gingival tumor
Caesarean section surgery	38 week		38 week 5 day
Newborn information	Female, 2875 g		Male, 3160 g
	birth smoothly, full of vitality, without any breathing difficulties or other abnormalities		
Clinical examination	• Maxillary • Pedicle and the lobulation is not particularly obvious • Due to the baby’s scratching, the surface of the tumor ruptures and bleeds, forming blood scabs		• Maxillary • Clearly lobulated, with a wider base and no obvious peduncle
Operation time	2nd day after delivery		Immediately after delivery
	Prevent pediatric patients from sucking their fingers and causing surface rupture and bleeding of the tumor		
Anesthesia	General anesthesia for tracheal intubation		
Surgery	• The pedunculated base is small • The wound was not sutured after electrocoagulation • hemostasis after resection		• Due to the lack of pedicle and wide base, the wound after resection is relatively large • Used 3 needles to reduce the size of the wound
Intraoperative observation	The tumor has no obvious blood supply vessels and is not connected to hard tissue		
Postoperative	Breastfeeding was performed on the second day after surgery, and the patients did not feel any discomfort		
Histopathological examination	• composed of large polygonal cells, cytoplasm filled with eosinophilic granules • small and round nucleus • negative S-100 protein		
Follow up	• All babys teeth erupted normally • Without any developmental abnormalities or deformities • The tumor did not recur		

### Antenatal preparation and delivery

Consultation composed of oral and maxillofacial surgeon, pediatrician, obstetrician and anesthesiologist was organized for both cases to make a considered treatment plan. Simultaneously, prenatal counselling was made to appease the anxiety of the parents. Considering that the oral mass might cause respiratory tract obstruction and bleeding during delivery, caesarean sections were opted for in both cases. The operations were conducted successfully at the Qilu Hospital Obstetrics Center.

### Postnatal examination

Both infants were stable during production, displaying a pink color and full of vitality, without any signs of respiratory distress or other complications. The first infant was a female, was born at 38 weeks of gestation and weighed 2875 g. The second one was a male and born at 38.5 weeks of gestation, with a weight of 3160 g. Clinical examination showed that the masses were pink, elastic and smooth-surfaced, originating from the mouth. The first lesion was non-lobular, pedunculated, and attached to the alveolar ridge of the right maxillary incisor. Because of scratching by the infant, the surface of the lesion bled and formed ulceration (Figure 1B). The second lesion was sessile and lobular, attached to the right maxillary alveolar ridge from the midline to the premolar tooth (Figure 2B). None of the lesions caused ventilation obstruction; however, both infants experienced difficulty maintaining adequate lip seal, leading to feeding difficulties.

### Surgical excision

After consulting with the pediatrician and anesthesiologist, surgical excisions were carried out under general anesthesia. Written consents from the patient’s guardians were obtained. The initial plan was to perform the first surgery a few days later to minimize risks. However, due to the ulceration and bleeding caused by scratching of the infant, the resection was performed on the following day. To prevent a recurrence, the second surgery was promptly performed after delivery. Both operations were performed under general anesthesia with tracheal intubation. Monopolar electrocautery was employed to minimize blood loss. Since the second lesion was sessile with a broad base, the wound was closed with 3 absorbable sutures (Figure 2D). Neither lesion was clearly defined or connected to hard tissue. Biopsy specimens were sent for histopathological examination to confirm the diagnosis. Breastfeeding was initiated on the second day of operation, and the children did not experience any discomfort.

### Histopathological examination

**FIGURE 3:**
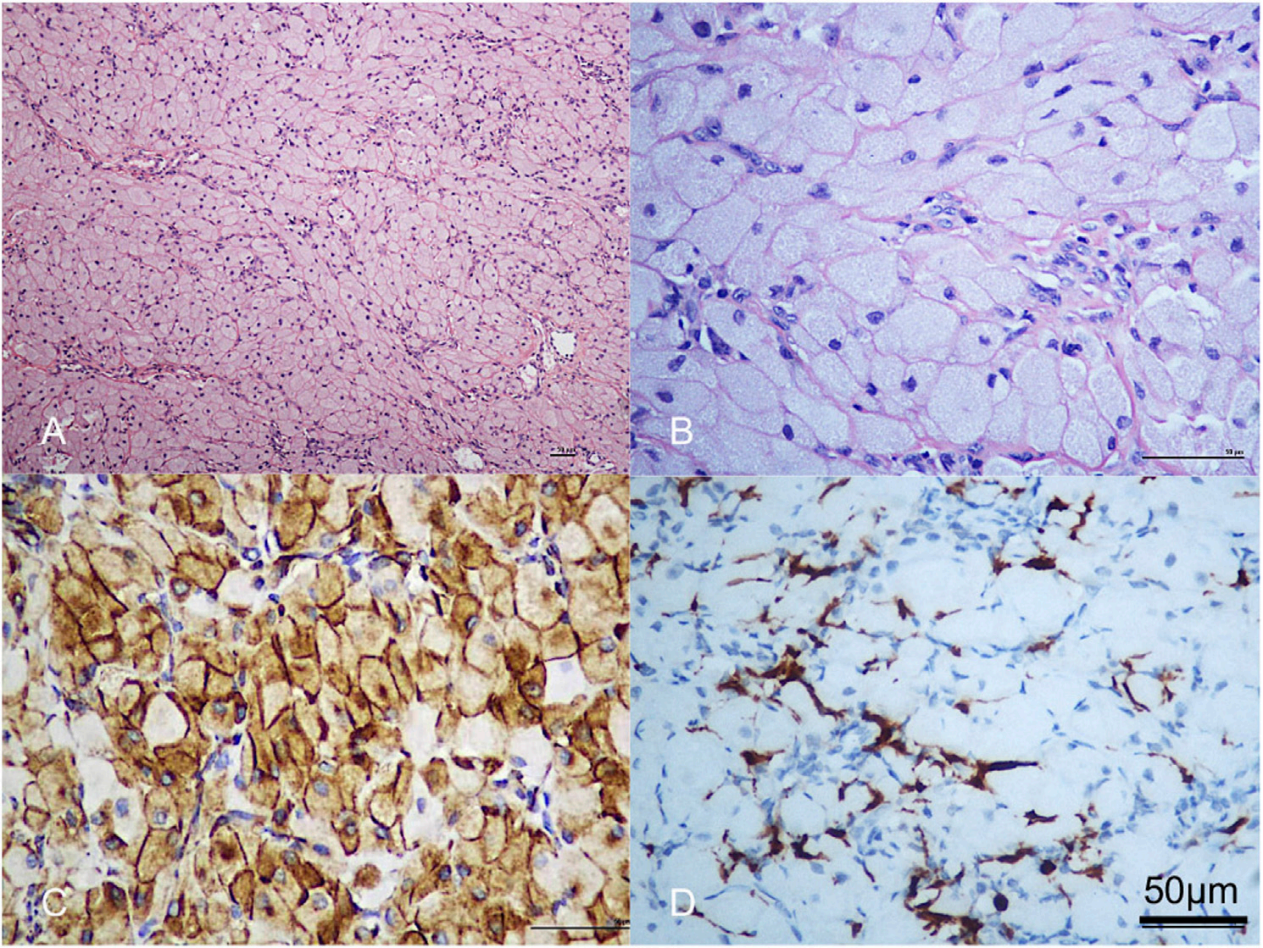
**(A,B)** Histologic sections showed the lesion was composed of large polygonal cells, filled with eosinophilic granules in the cytoplasm. The nucleus was small and round. Scale bar = 50 µm. **(C)** Vimentin was strongly positive. Scale bar = 50 µm. **(D)** The lesional cells were negative for S-100 protein; note dendritic cell staining. Scale bar = 50 µm.

The initial lesion’s specimen measured approximately 30 × 15 mm in size (Figure 1C), while the subsequent one was roughly 30 × 20 mm (Figure 2C). The cross-section was homogeneous, with a yellowish-white color, and there was no evidence of liquefaction, blood sinuses, or necrosis (Figure 1C up-right). Histopathological examination revealed that the lesion was composed of large polygonal cells filled with eosinophilic granules in their cytoplasm. The nuclei were small and round (Figures 3A, B). Immunohistochemical analyses demonstrated that Intracellular vimentin exhibited a positive staining, The lesional cells were negative for S-100 protein, take note of the dendritic cell staining. Neither NSE protein nor CD68 protein were detected (Figures 3C, D).

### Follow-up

The babies were discharged at the regular time, and the sutures were left untreated, allowing them to naturally fall off. Perfect wound healing was observed 2 weeks later. Follow-up visits were conducted every 6 months until all deciduous teeth emerged. All deciduous teeth had normal eruption and no dysplasia and deformity turned up. No recurrence was observed in either case. (Picture not shown)

## Discussion

CGCE was initially described by Neumann in 1871 [3]. CGCE exclusively occurs in newborns and exhibits features of spontaneous regression, lack of recurrence and malignant transformation. Thus, it is not considered to be a real neoplasm but rather a reactive hyperplasia [15, 16]. This condition is uncommon, with an incidence rate of 0.0006% [4, 5]. In our hospital, the two cases reported in this article mark the only occurrences in the past 20 years. The lesion commonly develops in the anterior alveolar ridge of the maxilla, particularly in the area of canine and lateral incisors (which is more frequent than in the mandible, with a 3:1 ratio) [6]. Approximately 10% of cases involve both the maxilla and mandible [8]. The lesion occurs more frequently in females than in males (8-10:1), which may be associated with the stimulation of endogenous hormones in the uterus [17]. The lesion is solitary, lobular, sessile or pedunculated, firm, and has a pink color with a smooth surface. The diameter of the lesion can vary from a few millimeters to tens of millimeters, with the largest reaching a diameter of 9 cm [18]. Small lesions generally have no impact on the infant, while larger ones may cause respiratory obstruction and difficulties with breastfeeding.

CGCE can be detected through prenatal obstetric ultrasound [19, 20]. The ideal time for prenatal ultrasound testing is typically during the third trimester, with no reports prior to the 26th week of gestation [21]. All of the current cases were identified at approximately the 34th week of gestation. Although previous ultrasound examinations were normal, the presence of lesions should not be assumed to be non-existent, as they may have been too small to detect. The presence of these lesion may affect fetal swallowing which could cause excessive amniotic fluid [8, 22]. While the two cases we reported here exhibited normal amniotic fluid, this may be attributed to the fetus’s ability to swell [14]. Although prenatal ultrasound observations have been obtained, the findings are nonspecific, making it difficult to make an initial diagnosis of CGCE. Differential ultrasonic diagnoses may include congenital malformations and intraoral tumors such as teratomas, hemangiomas, and lymphangiomas [22, 23]. As mentioned above, the primary prenatal impression of the mass was likely a congenital teratoma rather than CGCE. Nonetheless, prenatal ultrasound examination plays a crucial role in facilitating early communicating with parents and in alleviating parental anxiety. Furthermore,a multidisciplinary consultation can be organized to ensure a safe delivery and to devise a well-considered treatment plan.

The gross specimen of CGCE presents as a solid, smooth mass with a pink hue, which is congruent with the surrounding gums. Bang KO had documented a case where the tumor exhibited a red appearance due to the prominent blood vessels covering its surface [24]. The cross-section appears homogeneous, presenting as yellowish white or greyish, as previously reported. Histologically, CGCE is characterized as a benign tumor composed of large, polygonal cells with abundant eosinophilic, granular cytoplasm, small and round nuclei, and no mitotic figures. Currently, there are several theories regarding the origin of these lesions, including odontogenic, myogenic, epithelial, histiocytic, neural, and mesenchymal origins [6, 25–28]. However, there is still no consensus opinion. Currently, the majority of scholars support the theory of mesenchymal or neurogenic origin. Regarding immunohistochemistry, numerous documents have been reported, and the results have varied. Among the markers, a positive reaction for vimentin is the most frequently reported. Therefore, the majority of scholars believe that CGCE originates in mesenchymal cells [2, 12, 29, 30]. While others concur that the lesion is derived from nerve cells, based on the presence of NSE immunopositivity [11, 31, 32]. However, it’s worth noting that NSE is not specific to tumor cells, so this hypothesis must be further discussed. Additionally, according to one report, the presence of S-100 immunopositivity supports the notion that CGCE arises from Schwann cells and originates in nerve cells [14]. Nevertheless, most prior studies have yielded negative results for S-100, which contradicts the neural origin [25–30]. In the current study, the Immunohistochemical stains were strongly positive for vimentin and negative for NSE. These results support the hypothesis of mesenchymal origin. CGCE is typically diagnosed based on its clinical features, with spontaneous regression being a common one [15, 32]. One explanation for spontaneous regression is the stimulation of the tongue during the embryo stage and the sucking effect after birth, which can transform CGCE from a granular to a fibrous mass with the help of macrophages [12]. Additionally, maternal estrogen and fetal ovarian hormone levels may play a role. The absence of estrogen stimulation after birth leads to the tumor’s spontaneously regresses [17]. In addition to the aforementioned factors, it is important to consider the blood supply to the lesion. During the removal of the lesions, no significant supply vessels were identified. Consequently, as the growth environment changes, the blood supply to the lesion gradually diminishes, leading to spontaneous regression. Furthermore, the characteristic spontaneous regression of CGCE may contribute to its low incidence rate. Small lesions typically exhibit no symptoms and regress spontaneously after birth, often going unnoticed by both obstetricians and parents, resulting in a lack of case statistics. The primary differential diagnosis of CGCE is granular cell tumor (GCT) due to their comparable histological morphology, albeit with distinct clinical behaviors. GCT primarily affects the tongue of adults and may reappear post-resection, Whereas CGCE is exclusively observed in newborns and spontaneously regress without the risk of recurrence following removal [16]. Furthermore, GCT is derived from Schwann cells and exhibits positive S-100 reaction, while CGCE does not. Studies have indicated that cases with positive S-100 reaction should be diagnosed as GCT instead of CGCE [16]. However, we maintain that S-100 reaction should not be the sole diagnostic criterion, as histological and clinical characteristics, such as occurrence exclusively in newborns, spontaneous regression, and non-recurrence post-surgical removal, are adequate to differentiate GCT.

The treatment for CGCE encompasses observation for spontaneous regression and surgical resection. The spontaneous regression of CGCE has been documented in literature [20]. However, not all lesions undergo spontaneous regression, as Ritwik et al reported a case which did not regress after 6-month observation period [33]. Additionally, other scholars have also noted that tumor regression was not clearly evident [34, 35]. According to reports, lesions with a diameter of less than 2 cm are permissible to wait for spontaneous regression [33]. However, larger ones may cause interference with breastfeeding or respiration and necessitate removal. Some individuals opt to undergo the surgery several months after birth to ensure its safety [34]. In the two cases we reported, early and immediate operations were chosen for the following reasons: 1. The lesions were diagnosed prenatally, and a multidisciplinary consultation was organized to make a therapeutic plan. 2. The hospital’s perfect anesthesia technology for newborns ensured the safety of the operation. 3. Although there was no ventilation obstruction, breastfeeding difficulties appeared in both infants. 4. To prevent rupture and bleeding caused by the baby’s scratching. 5. To alleviate parents’ anxieties regarding breastfeeding difficulties, tooth eruption, local deformities, and ugly appearance. The operations were successfully completed with a short duration and minimal bleeding. The infants remained stable post-surgery and commenced breastfeeding the following day. Follow-up visits revealed normal tooth eruption, with no signs of tumor recurrence or local abnormalities.

## Conclusion

CGCE is a rare benign tumor that arises in newborns. Prenatal diagnosis is beneficial as it allows for prior communication with parents and reduces parental anxiety. Additionally, a multidisciplinary consultation can be arranged to ensure a safe of delivery and comprehensive management of the lesion. Early or immediate surgical intervention is advocated as it may lead to respiratory tract obstruction, feeding difficulties, infant discomfort and parental anxiety.

## Data Availability

The datasets presented in this study can be found in online repositories. The names of the repository/repositories and accession number(s) can be found in the article/Supplementary Material.
